# Does prescribing long acting antipsychotic injection increase mortality or morbidity in patients who continue to use illicit drugs?

**DOI:** 10.1192/j.eurpsy.2023.2293

**Published:** 2023-07-19

**Authors:** N. Sud

**Affiliations:** Cumbria Tyne and Wear NHS Foundation Trust, Newcastle upon Tyne, United Kingdom

## Abstract

**Introduction:**

Substance use disorders among individuals with psychotic disorders are a common. This is generally linked to more symptoms, worsened illness and high rates of treatment non-adherence. Long acting injections offer reliable drug delivery, reduce relapse risk and mortality (Khan et al 2016, Correll et al 2020) and can be used in individuals using illicit substances (Coles et al 2021, Erdogan et al 2021).

**Objectives:**

Aim was to look at literature comparing morbidity and mortality between oral versus long acting antipsychotics in patients with Schizophrenia and psychotic disorders who are currently using illicit drugs.

**Methods:**

A literature search was conducted using keywords long acting antipsychotic injection / depot and substance use on databases EMBASE, Psychinfo, Medline and CINAHL.

**Results:**

A review of psychopharmacological properties of first and second generation LAI (Taylor 2009) noted that use is complicated by adverse effects and confusion over dose response relationships. Atypical antipsychotics may induce direct cardiovascular alterations, probably through apoptotic effect of dopamine receptor D2 (DRD2) blockade. A cross sectional study (Dehelan et al 2021) looked at cardiac ejection fraction (EF) in 123 patients with Schizophrenia or Schizoaffective disorder on Aripiprazole, Olanzapine, Paliperidone and Risperidone Long acting injections. A trend was observed indicating that patients treated with an antipsychotic associated with a lower affinity for the DRD2, such as Olanzapine, have higher EF values than patients treated with antipsychotics with a stronger binding to the DRD2, such as Paliperidone and Risperidone. Patients receiving Aripiprazole, which has the strongest affinity for the DRD2 from all four antipsychotics but is also a partial DRD2 agonist, display higher EF values than those on Paliperidone and Risperidone.

A critical systematic review and meta-analysis of randomised long term trials looking at oral vs depot antipsychotic drugs for Schizophrenia (Leucht, Claudia et al 2011) included 10 studies. Relapse was significantly reduced in patients on long acting injections. There was limited data on non-adherence, drop outs and adverse events. This data revealed no significant differences. There is concern with methodological issues in trials and possibility of bias.

Another systematic meta review of randomised controlled trials of long acting antipsychotic injections (Adams Clive et al 2001), found no difference in adverse effects in long acting injections vs oral medications but small benefit on global outcome measure (relapse).

**Conclusions:**

Larger studies of populations of patients who are using illicit substances and are on long acting antipsychotic injections are required to discern differences in long term adverse effects in this population .

**Disclosure of Interest:**

None Declared

